# Development of *Dichelops furcatus* (Hemiptera: Heteroptera: Pentatomidae) Reared on Spring Cereals Versus Soybean

**DOI:** 10.1093/jisesa/iey102

**Published:** 2018-10-20

**Authors:** Antônio R Panizzi, Tiago Lucini, Taynara Possebom

**Affiliations:** 1 Laboratório de Entomologia, Embrapa Trigo, Passo Fundo, RS, Brasil; 2 Faculdade de Agronomia, Universidade de Passo Fundo, Passo Fundo, RS, Brasil

**Keywords:** stink bug, biology, feeding, preference, plant

## Abstract

The performance and preferences of the stink bug, *Dichelops furcatus* (F.), for spring cereals (wheat, rye, triticale, oat, and barley) were compared in the laboratory to their preferred host crop, soybean pods. Nymphs took significantly less time to reach adulthood on soybean pods compared to those fed seed heads of the five spring cereals tested. Wheat and rye yielded the longest developmental times, while nymphs fed triticale, oat, or barley developed faster, but still not as fast as those reared on soybean pods. On all foods ≥78% of nymphs reached adulthood. Adult body weight was significantly greater on soybean pods than on any of the spring cereals, and adults increased in body weight on all food sources tested. Fecundity was significantly greater for females fed soybean pods than those reared on the cereals. Egg viability was ≥66.9% except for bugs fed triticale (31.4%). In general, adult *D. furcatus* preferred soybean pods to seed heads of spring cereals, with wheat being preferred over the other spring cereals.

Stink bugs (Heteroptera: Pentatomidae) are crop pests worldwide (McPherson and McPherson 2000, Panizzi et al. 2000). Recently, various pentatomids have colonized new agroecosystems and proliferated as invasive pests (Panizzi 2015, McPherson 2018). Under no-till farming in the Neotropics, some stink bugs are able to reproduce year-round due to the availability of crop residues in fields (Panizzi 1997), sometimes resulting in drastic increases in pentatomid populations.

The ‘green-belly’ stink bug, *Dichelops furcatus* (F.), occurs in several South American countries, primarily in the so-called south-cone countries, including Argentina, Brazil, and Uruguay (Grazia et al. 2015). In southern Brazil during the 1970s this species was only an occasional pest of soybean, *Glycine max* (L.) Merrill (Fabaceae) (Galileo et al. 1977, Panizzi et al. 1977). More recently, however, *D. furcatus* is occurring in greater abundance on cultivated and non-cultivated plants (Chiaradia et al. 2011, Smaniotto and Panizzi 2015).

Cultivated host plants of *D. furcatus* include wheat, *Triticum aestivum* L. (Poaceae), during winter and spring in the Neotropics (Chocorosqui and Panizzi 2004); sunflower, *Helianthus annuus* L. (Asteraceae) (Frota and Santos 2007); and maize, *Zea mays* (L.) (Poaceae) (Roza-Gomes et al. 2011) during the summer. In addition, *D. furcatus* colonizes other spring cereals in southern Brazil such as oat, *Avena sativa* L. (Poaceae), during the vegetative (early plant development) and the reproductive periods (booting and seed-filling stage of the plant heads) (Pereira et al. 2013). On soybean, the main damage occurs during the pod-filling stage (Panizzi et al. 1977); conversely, on wheat and maize, severe damage occurs primarily on seedlings (Panizzi et al. 2016). However, on wheat, green-belly stink bugs damage the booting and/or milk-grain stages resulting in discolored seed heads, and undeveloped and malformed grain (Panizzi et al. 2016); eventually, this damage may be of concern to wheat growers.

In the Near East, *Aelia* spp. stink bugs and *Eurygaster* spp. shield bugs (Scutelleridae) are perpetual pests of cereals wheat and barley (*Hordeum vulgare* L. (Poaceae)) (Javahery 1996). Several pentatomids are wheat pest in the United States, mainly *Oebalus pugnax* (F.) and *Euschistus* spp. (Buntin and Greene 2004, Koch et al. 2016).

*Dichelops furcatus* damages spring cereals in south-cone countries, yet information is lacking regarding its impact, and on its plant preferences. This dearth of information led us to conduct the present research. The objectives of this research were: 1) to evaluate the effect of reproductive structures of different spring cereals on nymphal development, survivorship, and body weight; 2) to evaluate the effect of these food sources on adult reproduction, survivorship, and body weight; and 3) to evaluate preferences of the bugs for the reproductive structures of the cereals tested versus soybean pods.

## Materials and Methods

### Laboratory Stink Bug Colony

Adults of *D. furcatus* were collected at the Embrapa wheat field station, located in Passo Fundo, Rio Grande do Sul, Brazil (28°15′S, 52°24′W). Adults, collected from the soil surface under crop residues in early spring 2017), were transported to the laboratory for rearing. Cages (25 × 20 × 20 cm) with filter paper in the bottoms were provided as needed with fresh green bean pods, *Phaseolus vulgaris* L., raw shelled peanuts, *Arachis hypogaea* L. (Fabaceae), and mature seeds of soybean. Cages were kept in a walk-in chamber maintained at 25 ± 1°C and 65 ± 5% RH, with a photoperiod of 14:10 (L:D) h hourly cycle. Cotton balls were provided as an oviposition substrate, and egg masses were collected daily. Collected egg masses were placed inside plastic boxes (11 × 11 × 3.5 cm) with filter paper in the bottom. Nymphs were reared to adulthood as described above. Egg masses obtained from the laboratory-reared adults were used for the nymphal study. As additional nymphs reached the adult stage, they were used for the adult study.

### Greenhouse Host Plants

Seeds of the different spring cereals and, for comparison, of soybean (pod-filling stage) since soybean is the main crop host exploited by *D. furcatus*), were seeded in pots (2L) maintained in a greenhouse. The following cultivars were used: wheat, ‘BRS Reponte’; oat, ‘BRS Neblina’; barley, ‘BRS Quaranta’; rye, *Secale cereale* L. ‘BRS Serrano’; triticale, *Triticosecale semisecale* (Mackey) K. Hammer & Filat ‘BRS Resoluto’; and soybean, ‘BRS 5601 RR’. For the nymph and adult studies (described below), the spring cereals were used at the R11.1 stage (milk-grain stage) (Large 1954); soybean plants were used at the R5 stage (pod-filling stage) (Fehr et al. 1971). Plants were grown using a standard oxysoil prepared for greenhouse studies at Embrapa. For the spring cereal plants, potted plants were placed under a shaded area, and a blower was used to provide cooler air (ca. 22°C); soybean plants were cultivated in a sunnier and warmer compartment of the greenhouse (ca. 28°C).

### Nymphal Development Study

#### Developmental Time and Survivorship

This study was conducted from December 2017 to January 2018. On the first day of the second instar (first instars aggregate and do not feed—but see Esquivel and Medrano 2014), nymphs were placed in plastic boxes (11 × 11 × 3.5 cm) lined with filter paper. The following food sources were tested: seed heads at milk-grain stage for wheat, oat, barley, rye, and triticale; and pods at the grain filling stage for soybean. For each food source, 10 nymphs were placed inside a respective box and replicated five times, totaling 50 nymphs/food source. One seed head or one piece of soybean stem with three pods were placed in each box, and stems were wrapped with wet cotton to prevent desiccations. The food and filter paper in boxes were changed every 2 d. The test boxes were placed at random in an environmental chamber maintained at 25 ± 1°C and 65 ± 5% RH with a photoperiod of 14:10 (L:D) h hourly cycle. Daily observations were made on molting and mortality. Total nymphal developmental time and total percentage mortality (from second instar to adult) were calculated.

#### Fresh Body Weight at Adult Emergence

As nymphs reached the adult stage, each adult was placed in a small plastic cup and had its body weight recorded using an electronic balance (Mettler Toledo MS 3002S/A01, Barueri, SP, Brazil). Mean body weights were calculated for females and males obtained from each food source.

### Adult Development Study

#### Adult Survivorship and Reproduction

This study was conducted from December 2017 to January 2018. Adults were obtained from nymphs reared in the laboratory using the foods described for maintaining the stink bug colonies. On the day of emergence, 60 pairs were separated and, each bug was weighed; this was done to allow calculation of subsequent body weight change (see below). The female/male pairs were formed at random, and each pair was placed in a plastic rearing box (11 × 11 × 3.5 cm) lined with filter paper. Adult performance was evaluated on the following foods (*n* = 10 pairs for each food): seed heads at milk-grain stage for wheat, oat, barley, rye and triticale, and pods at the filling stage for soybean. Each test box was provided with a single seed head of each cereal or a piece of soybean stem with three pods and maintained as described previously for nymphs. Daily observations were made to determine survivorship up to 40 d, and reproduction (% females that laid eggs, pre-oviposition time, egg masses and total eggs laid, and egg fertility). If a female or a male died before day 40, they were not replaced.

#### Fresh Body Weight

Adults (females and males) had their weight measured weekly for 4 wk. The percent fresh adult body weight changes of females and males over time were calculated weekly and compared during each week and cumulatively over the total time (4 wk). For analysis, fresh body weight change was calculated pooling the data for females and males.

#### Preferences for Plant Reproductive Structures

To test the preferences of *D. furcatus* for different reproductive structures of the spring cereals and soybean, dual comparisons (i.e., two different plant species) were conducted using cages (30 × 30 × 30 cm) with bottoms lined with filter paper. Four vials (10.0 × 4.5 × 2.0 cm diameter) (i.e., two for each food source were compared) containing water and short stems with seed heads (at the milk-grain stage) of the respective spring cereals or short branch of soybean with pods (at the pod-filling stage) were placed alternatively in the corners of each cage. Vials were topped with aluminum foil to avoid water evaporation. The following comparisons were tested: soybean pods versus seed heads of each one of the spring cereals studied (wheat, rye, barley, oat, and triticale); and seed heads of wheat versus those of the remaining cereals (rye, barley, oat, and triticale).

One adult *D. furcatus* (either female or male) was released in the center of each cage. After 24 h, twice daily observations were taken (9:00 a.m. and 4:00 p.m.) for 5 d, and the location of the bugs on one of the seed heads of cereals or pods of soybean were recorded. Each dual comparison was replicated four times (four cages). The number of observations per comparison totaled 40 (4 cages × 2 observations × 5 d). The percentage values of the preferences for the reproductive structures tested were calculated.

### Data Analysis

The analyzed variables for nymphs (developmental time and fresh body weight at adult emergence) and adults (reproductive performance and body weight change) were previously submitted to the Bartlett test using the ‘Bartlett.test’ function in the R software (R Development Core Team 2016) to determine the homogeneity of variances. Data were then transformed as necessary, to fulfill the normality distribution requirement prior to the analysis of variance (ANOVA), using an appropriate transformation for each case: √(x) for counting data, and arcsine √(x/100) to percentage data.

Data for female body weight at adult emergence were transformed, whereas males data were not, because they were normally distributed. Comparisons were done within each sex among all foods, therefore, any possible effect of female’s transformed data on male’s untransformed data was avoided, since no comparisons between sexes were performed.

In the sequence, dataset from each analyzed variable were fit in a one-way analysis of variance model using the ‘aov’ function in R, in which the dependent variable was the analyzed variable and the independent variable was the food sources tested. When applicable, means separations were done using the Tukey’s test, at *P* < 0.05, using the ‘TukeyC’ package (Faria et al. 2018). Data on the bugs preference for reproductive structures of plants were separated using the Pearson’s chi-square test (χ^2^) using the ‘chisq.test’ function in the R software.

## Results

### Nymphal Development

#### Developmental Time and Survivorship

**Fig. 1.**
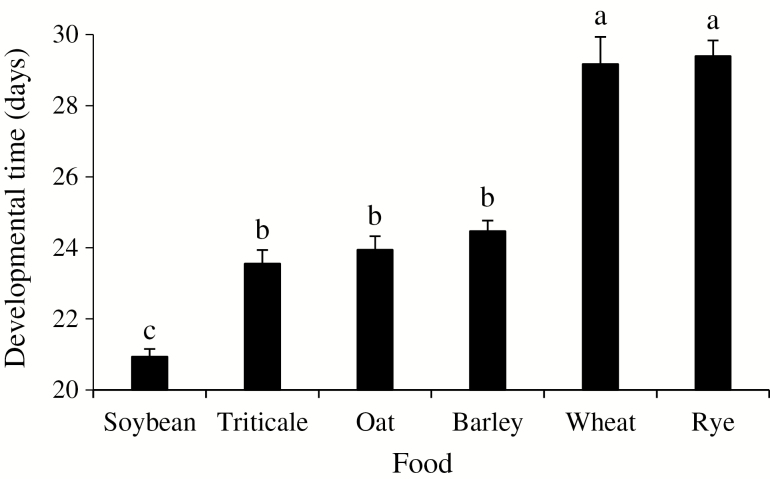
Developmental time of *Dichelops furcatus* nymphs feeding on seed heads of spring cereals and pods of soybean in laboratory conditions. Means (±SE) followed by the same letter are not significantly different using Tukey’s test, *P* < 0.05.

Total nymphal developmental time (second instar to adult) was variable (21–29 d) depending on the food source (Fig. 1). Nymphs took significantly less time to reach adulthood on soybean pods compared to those on the seed heads of the five spring cereals (*F* = 59.45; df = 5, 261; *P* < 0.001). Among them, seed heads of wheat and rye required the longest times for development, while nymphs fed triticale, oat, or barley developed faster, but still not as fast as those reared on soybean pods.

Despite the significant differences of developmental time between treatments, *D. furcatus* nymphs were able to reach adulthood on all foods tested. Survivorship of nymphs was variable, from the minimum of 78% on seed heads of oat to the maximum of 98% on soybean pods. On triticale and barley, nymphal survivorship was 94%, decreasing to 86 and 84% on seed heads of rye and wheat, respectively.

#### Fresh Body Weight at Adult Emergence

**Fig. 2.**
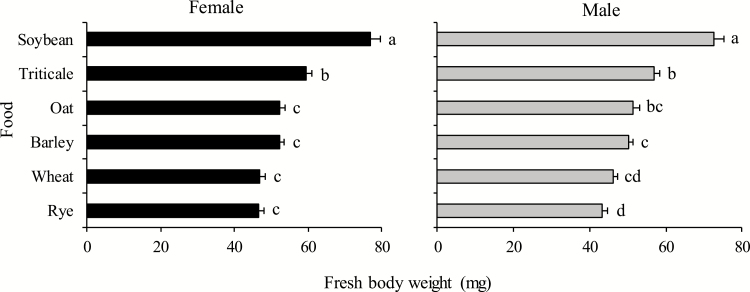
Fresh body weight of females and males *Dichelops furcatus* at the first day of the adult life feeding on seed heads of spring cereals and pods of soybean in laboratory conditions. Means (±SE) followed by the same letter in each gender are not significantly different using Tukey’s test, *P* < 0.05. Original data presented for females [for analysis, data were transformed to √(x)].

Body weight on the first day of adulthood was also variable according to the different foods tested. As for nymphs, females and males achieved significantly greater weight when fed on soybean pods than when fed seed heads of any of the spring cereals (females: *F* = 41.86; df = 5, 112; *P* < 0.001; males: *F* = 50.52; df = 5, 143; *P* < 0.001) (Fig. 2). Among them, significantly less fresh body weight-gain occurred for females on seed heads of oat, barley, wheat, and rye; weight did not differ among these cereal sources, and these weights were lower than for the triticale treatment. For males, a similar situation was observed, with wheat and rye yielding the lower weight-gain compared to that for triticale and oat treatments, with the barley treatment being intermediate (Fig. 2).

### Adult Development

#### Adult Survivorship and Reproduction

**Fig. 3.**
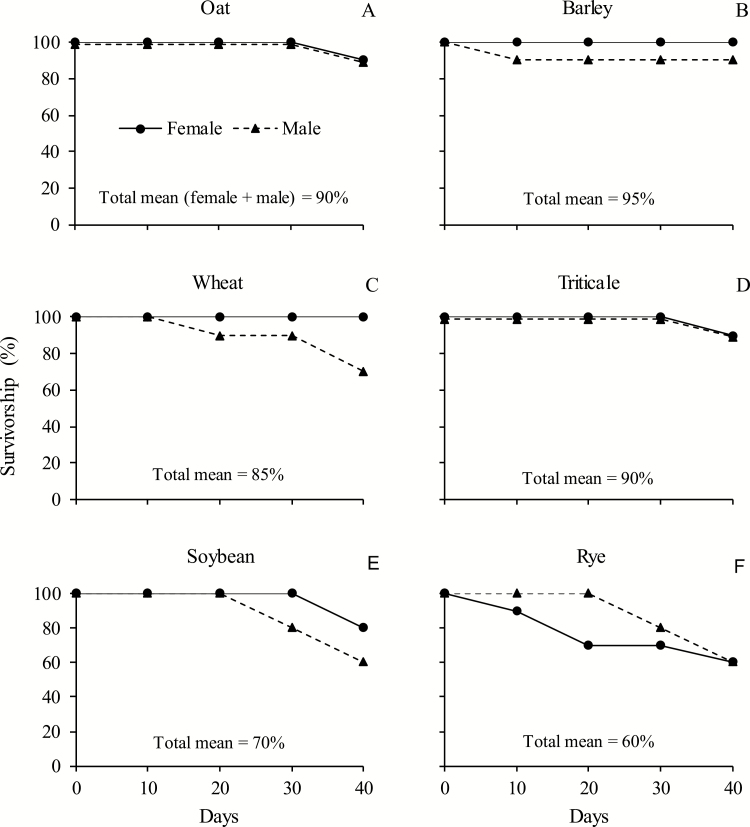
Survivorship (%) of adult females and males *Dichelops furcatus* (*n* = 10) up to 40 d feeding on seed heads of spring cereals and pods of soybean in laboratory conditions. Seed heads of oat (A), barley (B), wheat (C), triticale (D), and rye (F); and pods of soybean (E).

At 40 d of adult life survivorship for both *D. furcatus* females and males remained ≥85% on most food sources tested (Fig. 3). On seed heads of rye survivorship for both sexes was the lowest (60%). On soybean pods percent survivorship was intermediate (70%). In general, survivorship values for females and males were similar, although survivorship for males tended to be lower, except on rye, for which the opposite was observed (Fig. 3).

The reproductive performance of *D. furcatus* was variable according to the food source utilized. Oviposition varied from 30% (on rye and triticale) to 80% (on oat) (Table 1). Pre-oviposition time was significantly shorter (about half) on soybean pods, compared to that observed for seed heads of most spring cereals tested (*F* = 5.53; df = 5, 23; *P* < 0.01). Fecundity, in terms of total egg masses deposited, was significantly greater (two to seven times) on soybean pods than on the seed heads of the spring cereals (*F* = 15.82; df = 5, 23; *P* < 0.001); the lowest number of egg masses laid were observed for the barley and on triticale treatments (≤3.4 masses). A similar situation was observed for the total number of eggs laid; females on soybean pods laid significantly more eggs (two to seven times more) compared to those fed on seed heads of cereals (*F* = 11.99; df = 5, 23; *P* < 0.001). Egg fertility was, in general, high (≥66.9%), except for those females fed on seed heads of triticale (ca. 30%) (Table 1).

**Table 1.** Reproductive performance of adult female *Dichelops furcatus* feeding on seed heads of spring cereals and pods of soybean in laboratory conditions

Food	% females laying eggs	Pre-oviposition time (d)^*a*^	Egg masses *(n*)	Number of eggs	Egg hatch (%)^*b*^
Soybean	50 (10)^*c*^	11.6 ± 0.4b	16.8 ± 1.8a (84)	218.0 ± 26.4a	79.0 ± 7.2a
Oat	80	24.1 ± 1.7a	7.9 ± 0.9b (63)	112.5 ± 14.2b	86.8 ± 2.2a
Wheat	50	21.4 ± 1.8a	4.6 ± 1.2b (23)	62.8 ± 16.6b	86.9 ± 2.9a
Rye	30	20.0 ± 6.0ab	5.0 ± 2.3b (15)	65.0 ± 32.7b	66.9 ± 14.7ab
Barley	50	22.6 ± 2.1a	3.4 ± 0.9b (17)	49.2 ± 13.9b	71.5 ± 13.2ab
Triticale	30	22.0 ± 2.0a	2.3 ± 0.7b (7)	32.0 ± 9.0b	31.4 ± 23.6b

Means (±SE) followed by the same letter in columns (except for % females laying eggs) are not significantly different using Tukey’s test, *P* < 0.05.

^*a*^Original data presented [for analysis, data were transformed in √(x)].

^*b*^Original data presented [for analysis, data were transformed in arcsine √(x/100)].

^*c*^Value within brackets indicates the initial number of females per food source.

#### Fresh Body Weight

**Fig. 4.**
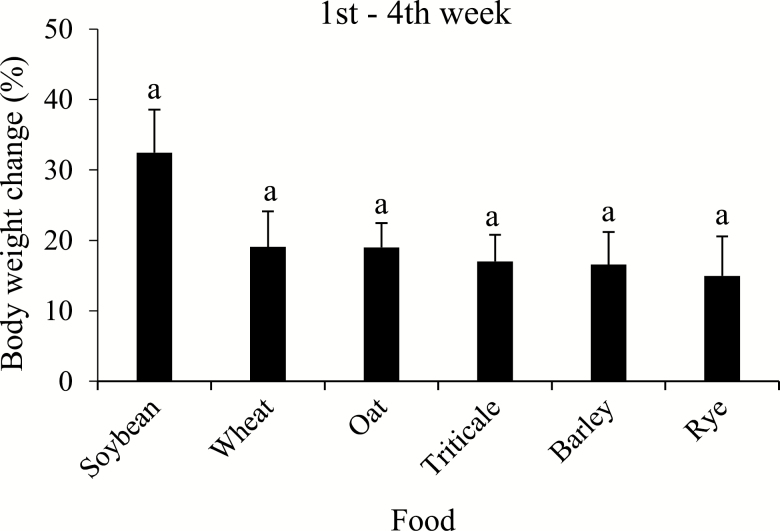
Fresh body weight change (%) of adult (female + male) *Dichelops furcatus* after 4 wk of adult life feeding on seed heads of spring cereals and pods of soybean in laboratory conditions. Means (±SE) followed by the same letter are not significantly different using Tukey’s test, *P* < 0.05.

The change in fresh body weight during adult life was variable on the different food sources utilized. During the first week, in general, adults (females and males) significantly increased more weight on soybean pods compared to most of the cereal treatments (*F* = 12.52; df = 5, 113; *P* < 0.001); however, on seed heads of oat, adults significantly lost body weight. During the following 3 wk, body weight-gain was minimal on all foods, except on seed heads of oat, for which the body weight lost during the first week was recovered (Table 2). At 4 wk, adults increased in body weight on all food sources tested, and did not significantly differ among treatments (*F* = 1.70; df = 5, 106; *P* = 0.14). However, weight-gain tended to be greater for bugs fed soybean pods (Fig. 4).

**Table 2.** Fresh body weight change (%) of adult (female + male) *Dichelops furcatus* during the first 4 wk of adult life feeding on seed heads of spring cereals and pods of soybean in laboratory conditions

Food	First week^*a*^	Second week^*a*^	Third week^*a*^	Fourth week^*a*^
Soybean	26.9 ± 4.1aA (20)^*b*^	2.2 ± 1.6aB (20)	0.4 ± 1.1aB (20)	1.0 ± 1.2aB (18)
Rye	11.3 ± 4.7bA (20)	−0.5 ± 3.3aA (19)	0.9 ± 2.7aA (17)	2.2 ± 3.1aA (16)
Barley	10.7 ± 3.2abA (20)	3.7 ± 3.2aAB (19)	6.8 ± 2.5aA (19)	−4.9 ± 2.5aB (19)
Triticale	9.5 ± 4.0bA (20)	4.1 ± 3.3aA (20)	3.1 ± 2.6aA (20)	2.0 ± 2.8aA (20)
Wheat	6.1 ± 2.8bA (19)	7.3 ± 3.3aA (19)	5.3 ± 3.3aA (19)	0.7 ± 2.9aA (19)
Oat	−10.1 ± 2.4cB (20)	11.9 ± 3.4aA (20)	12.4 ± 3.7aA (20)	7.9 ± 2.8aA (20)

Means (±SE) followed by the same lowercase and uppercase letters in columns and rows, respectively, are not significantly different using Tukey’s test, *P* < 0.05.

^*a*^Original data presented [for analysis, data were transformed to arcsine √(x/100)].

^*b*^Value within brackets indicates the number of adults (female + male).

#### Preferences for Reproductive Structures

**Fig. 5.**
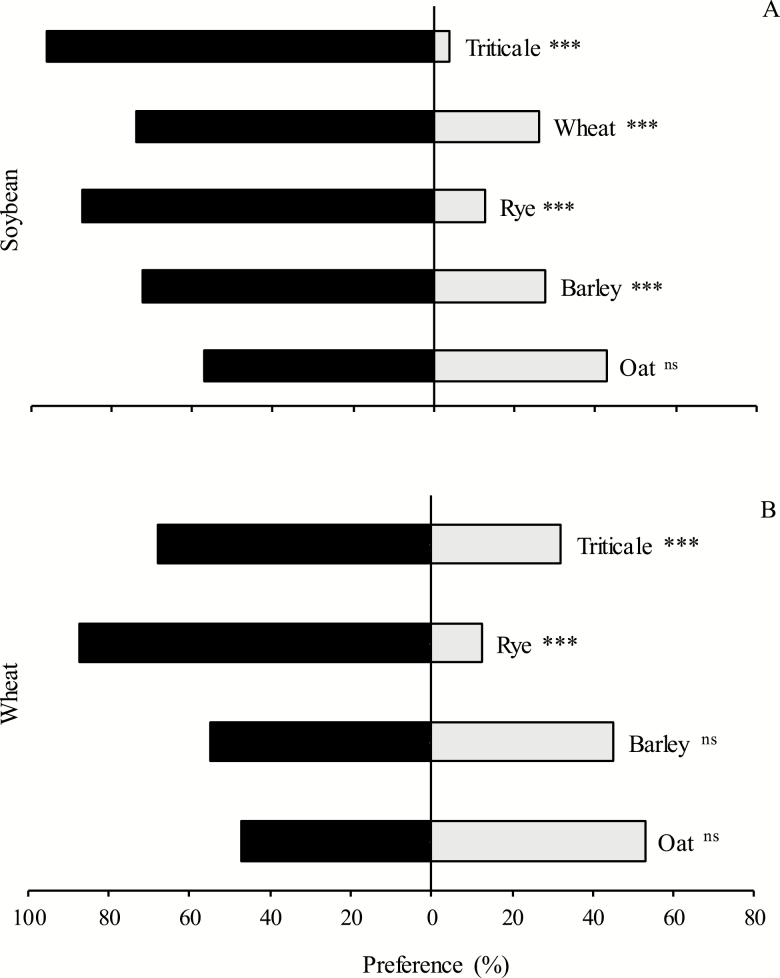
Preference ratios (%) of *Dichelops furcatus* on different combinations between pods of soybean and seed heads of spring cereals (A) and between seed heads of wheat and seed heads of other spring cereals (B). ns, nonsignificant different and ***significant different (*P* < 0.001) using Pearson’s chi-square test (χ^2^).

On the reproductive structures of plants, with dual preference tests conducted, *D. furcatus* adults preferred soybean pods compared to most seed heads of spring cereals ${\text{χ}}_{\text{calc}}^{2}=\text{84}.\text{6}$ against triticale; ${\text{χ}}_{\text{calc}}^{2}=23.0$ against wheat; ${\text{χ}}_{\text{calc}}^{2}=54.8$ against rye; ${\text{χ}}_{\text{calc}}^{2}=19.4$ against barley, all *P* < 0.001), except for oat seed heads ${\text{χ}}_{\text{calc}}^{2}=2.00$*P* = 0.161) (Fig. 5A).

When the preference tests were run comparing the seed heads of wheat against the seed heads of the remaining spring cereals, the former was preferred compared to seed heads of triticale ${\text{χ}}_{\text{calc}}^{2}=13.0$*P* < 0.001), or rye ${\text{χ}}_{\text{calc}}^{2}=54.8$*P* < 0.001). However, no significant differences were obtained when seed heads of wheat were compared to those of barley ${\text{χ}}_{\text{calc}}^{2}=1.00$*P* = 0.317) or oat ${\text{χ}}_{\text{calc}}^{2}=0.36$*P* = 0.548) (Fig. 5B).

## Discussion

We demonstrated that, in addition to soybean, the green-belly stink bug, *D. furcatus*, can develop and reproduce when fed seed heads of the common spring cereals grown in South America. Nevertheless, nymphs fed on soybean pods matured more quickly and were heavier than those reared on the spring cereals tested, which was to be expected since soybean is the principal crop food-plant for *D. furcatus* (see references in Smaniotto and Panizzi 2015). Because we used legumes (fresh green bean pods, raw shelled peanuts, and mature seeds of soybean) to rear the bugs one could argue that this might have preconditioned the bugs to perform better on soybean. However, in insects the argument of preimaginal (nymphal) food host-choice conditioning, known as Hopkins’ host-selection principle, is controversial (Barron 2001). On the spring cereals, the delay in nymphal development and lower body weight at adult emergence points to differences in chemical (nutritional quality and or presence of allelochemicals) and/or physical attributes (e.g., variable size and anatomy of seed heads; see Li et al. 2010) of cereal versus legume host plants. However, nymphal survivorship on the cereals tested (≥78%) was surprisingly high compared to that for other pentatomid pests of legumes (Panizzi 1997).

To date, there has been little research conducted on the feeding habits of *D. furcatus*; most existing data for *Dichelops* is for the similar congeneric species, *D. melacanthus* (Dallas). In general, nymph developmental time of the latter species on seeds/fruits of different plants fell within the range observed in the present study (21 to 29 d) (Chocorosqui and Panizzi 2002, 2008; Panizzi et al. 2007; Silva et al. 2013; Bortolotto et al. 2016). Exceptions are on immature soybean pods plus mature seeds (shorter time – ca. 18 d – Chocorosqui and Panizzi 2003), on seed heads of wheat (longer time – ca. 33 d – Chocorosqui and Panizzi 2008), and on seeds of tropical spiderwort, *Commelina benghalensis* L. (even longer – ca. 37 d – Silva et al. 2013).

Nymphal survivorship for *D. furcatus*, which varied from 78 to 98%, were generally higher that reported for *D. melacanthus* fed on various food sources, as follows: 23–40% on seed heads of wheat (Chocorosqui and Panizzi 2008); 56 and 70% on immature soybean pods plus mature seeds (Chocorosqui and Panizzi 2002, 2003, respectively); 55% on immature soybean pods (Panizzi et al. 2007), and 73 and 68% on immature maize ears (Panizzi et al. 2007; Bortolotto 2016, respectively).

These developmental and survivorship variations mentioned above for *Dichelops* nymphs may be due to the different food sources and/or rearing methods (e.g., nymphs reared singly or in groups). In our study, nymphs were raised in groups and, in part, this may explain the higher survivorship obtained as compared to other similar studies in which nymphs were raised singly. Phytophagous heteropterans reared in groups usually perform better than when reared singly in the laboratory (e.g., Ralph 1976, Panizzi et al. 2005).

It is interesting to note that, except on soybean, nymphs of *D. furcatus* are seldom found in the field colonizing the spring cereals tested. Apparently, adults do not oviposit on them at the time cereal plants produce seed heads, and prefer to colonize other crops available in the area, such as canola, *Brassica napus* L. (Brassicaceae) (Marsaro Jr. et al. 2017). Adults of *D. furcatus*, although polyphagous, may not recognize the spring cereals as suitable hosts.

The fact that the majority (≥60%) of *D. furcatus* survived past 40 d of adult life fed on the reproductive structures of spring cereals tested and on soybean, were able to lay eggs, and gain body weight demonstrates that these food sources are potentially important to their phenology. For example, adult survivorship of the congeneric *D. melacanthus* on seed heads of wheat, past 40 d, was ca. 50% for both females and males (Chocorosqui and Panizzi 2008), lower than the values we obtained for *D. furcatus* on seed heads of wheat (100% for females and 70% for males).

Fecundity, which was variable among the food sources tested, was greater on soybean pods, as to be expected from the known phenology of *D. furcatus*. This is similar to the results obtained with *D. melacanthus* fed on soybean pod/ immature seeds where egg masses were laid almost five times more compared to females fed on seed heads of wheat (Chocorosqui and Panizzi 2008). All spring cereals were considered less suitable and, although we could not find a clear explanation, these food sources may possess detrimental factors or poorer nutrition. In fact, they were less preferred (except oat) compared to soybean.

In summary, stink bugs seem to be expanding worldwide. They are colonizing crops such as soybean and maize (Koch and Pahs 2014, 2015), and winter and spring wheat (Koch et al. 2016) in areas as far north as Minnesota where they were seldom observed in the past. These associations may occur only sporadically and bugs may not reach pest status on these crops in such areas, but these associated plants may play an important role to their phenology. Therefore, our results conducted with *D. furcatus* in the Neotropics presents a similar situation to observations in northern temperate regions.

In conclusion, these laboratory studies coupled with field observations indicate that the spring cereals tested, i.e., wheat, oat, barley, rye, and triticale, during the reproductive period (presence of seed heads) provide a potential alternate food source for the green-belly stink bug, *D. furcatus*. Nymphs were able to complete development when feeding on them and adults reproduced and gained body weight in the laboratory. However, in the field, populations do not yet seem to have attained economically damaging levels. This fact led us to conclude that these spring cereals serve as alternate hosts mostly to sustain populations that will subsequently colonize summer crops, such as soybean, which is known as a preferred host plant and upon which *D. furcatus* could potentially reach pest status.
